# Evaluating the Efficacy of Neurofeedback in Post-Bariatric Surgery Patients: A Pilot Study

**DOI:** 10.3390/jpm15100454

**Published:** 2025-09-29

**Authors:** Claudia Scaramuzzino, Clara Lombardo, Giulia Esposito, Maria Rosaria Anna Muscatello, Antonio Bruno, Marco Populin, Giuseppe Navarra, Fabio Guccione, Carmela Mento

**Affiliations:** 1 Department “Scienze Della Salute”, University of Catanzaro, 88100 Catanzaro, Italy; claudia.scaramuzzino@studenti.unicz.it; 2 Department of Engineering, University of Messina, 98166 Messina, Italy; giu.espo23@gmail.com; 3 Department of Biomedical and Dental Sciences and Morphofunctional Imaging, University of Messina, 98125 Messina, Italy; mmuscatello@unime.it (M.R.A.M.); antonio.bruno@unime.it (A.B.); cmento@unime.it (C.M.); 4 S.S. C.S.M. 24 H San Vito al Tagliamento-D.S.M. A.A.S. n.5 Friuli Occidentale, 33078 San Vito al Tagliamento, Italy; marco.populin@asfo.sanita.fvg.it; 5 Department of Adult and Development Age Human Pathology “Gaetano Barresi”, University of Messina, 98122 Messina, Italy; gnavarra@unime.it (G.N.); guccione.fabio@libero.it (F.G.)

**Keywords:** neurofeedback, obesity, emotional eating, mental health, body image concerns

## Abstract

**Background**: Obesity remains a major global health challenge, and a significant proportion of bariatric surgery patients continue to experience dysfunctional emotional eating and body image concerns after surgery. Neurofeedback training (NFT) has been investigated as a potential intervention for maladaptive eating behaviours, but evidence in post-bariatric populations is still limited. **Methods**: Thirty-six patients who underwent sleeve gastrectomy were included, divided into an NFT group (N = 18) and a control group (N = 18). Assessments were performed at baseline and after 10 NFT sessions, using the Eating Disorder Inventory (EDI) and the Body Uneasiness Test (BUT). The intervention aimed to enhance alpha and theta waves with real-time feedback. **Results**: Compared with the control group, the NFT group showed significant improvements; specifically, reductions were observed in EDI subscales such as Drive for Thinness (*p* = 0.023, d = 0.51), Bulimia (*p* = 0.008, d = 0.92), Body Dissatisfaction (*p* = 0.015, d = 0.52), Ineffectiveness (*p* = 0.002, d = 0.89), Perfectionism (*p* = 0.006, d = 0.70), Interpersonal Distrust (*p* = 0.008, d = 0.82), and Interoceptive Awareness (*p* = 0.001, d = 0.91). Significant reductions were also found in BUT subscales including Weight Phobia (*p* = 0.041, d = 0.84), Body Image Concern (*p* = 0.039, d = 0.90), Avoidance (*p* = 0.027, d = 0.83), Compulsive Self-Monitoring (*p* = 0.013, d = 0.83), and Depersonalisation (*p* = 0.033, d = 0.85). **Conclusions**: The data indicate that NFT may help reduce emotional eating and related psychological factors in post-bariatric patients in the short term. However, studies with larger samples and longer follow-ups are needed to confirm its effectiveness and assess its clinical applicability.

## 1. Introduction

Obesity represents one of the most serious health challenges of our time, with significant implications for individual and collective health. It is related to a wide range of chronic diseases, including cardiovascular disease, type 2 diabetes, hypertension and some cancers, making it an independent risk factor for mortality in many developed nations [1]. Statistics suggest that the prevalence of obesity has reached epidemic proportions, affecting quality of life and health systems [2]. In adults, the Body Mass Index (BMI) is commonly used as an indicator of body fat, with values between 25 and 29.9 kg/m^2^ indicating overweight, while a BMI of 30 kg/m^2^ or higher is used to define obesity [3]. In this context of a significant increase in the obese population, it is crucial to understand the underlying mechanisms of obesity, as well as the psychological and behavioural factors that facilitate its progression. Among the factors associated with obesity, difficulties in managing emotions and the prevalence of negative emotions emerge as key elements in its genesis and maintenance. The literature defines emotional eating (EE) as the tendency to overconsume food in response to negative emotional states, such as anxiety, sadness, stress or anger, rather than in response to physiological hunger [4]. This behaviour involves a shift in attention from internal emotional experience towards immediate external stimuli, particularly food, with the aim of reducing self-awareness and regulating affect. This mechanism is consistent with the escape theory of self-awareness proposed in previous study [5], which suggests that individuals who resort to food to manage their emotions tend to divert their attention away from their negative emotional states, focusing instead on immediate stimuli in the environment, such as food. Emotional eating, therefore, represents a form of dysfunctional coping strategy, in which food intake has the function of alleviating emotional distress rather than satisfying an energy requirement [6]. Higher levels of emotional eating have been associated with higher BMI, indicating a direct link between this behaviour and the risk of obesity [7].

These dysfunctional behaviours not only complicate weight management but may also negatively influence perceived self-efficacy in the context of weight loss. One strategy often considered to bring about significant changes in weight loss in patients with severe obesity is bariatric surgery. Bariatric surgery has proven to be an effective option, leading to significant body weight reduction and improvements in associated comorbidities [8]. Bariatric surgery is considered an effective therapeutic intervention for the treatment of severe obesity characterised by BMI ≥ 40 kg/m^2^ or ≥35 kg/m^2^ in the presence of comorbidities. Several techniques, such as gastric bypass and sleeve gastrectomy, significantly reduce stomach capacity and limit nutrient absorption, leading to substantial weight loss. An additional study, Ref. [9] demonstrated that bariatric surgery can result in a body weight reduction of between 20% and 30% of the initial weight within the first 12–18 months post-operation. Furthermore, bariatric surgery is associated with improvements in obesity-related comorbidities, such as type 2 diabetes, hypertension and sleep apnoea, thus contributing to a better quality of life and reduced mortality [10]. These results highlight the importance of bariatric surgery as a therapeutic option in the management of obesity and its associated complications. However, it is important to recognise that some patients may not achieve the expected level of weight loss or may experience significant weight regain in the following decade. Outcomes may vary widely between individuals, and some patients may require additional interventions or support to maintain their results [11]. In particular, recent evidence indicates that even after surgery, a proportion of patients continue to present maladaptive eating behaviours and difficulties in emotional regulation, which may compromise long-term weight maintenance [12]. This underlines the need for complementary interventions specifically targeting psychological processes in the post-surgical phase.

As an alternative methodology in the treatment of patients with obesity, NeuroFeedback Training (NFT) is included, which represents a promising brain training methodology that relies on neurophysiological principles to offer a non-invasive approach to the control and modulation of brain activity [13,14,15,16,17,18]. Specifically, during the training process, the neurofeedback system analyses specific neural parameters, while a computational interface provides the patient with a continuous stream of real-time information about their physiological brain activity (e.g., through visual and/or acoustic signals). The patient is then called upon to self-regulate these parameters, receiving immediate feedback indicating whether or not the training goals have been achieved [15,17,19,20]. The scientific literature has documented the complex neurobiological processes that regulate eating behaviour, evidencing the central role of specific brain circuits in the modulation of appetite, food reward and inhibitory control [21]. Neuroimaging studies and research on the neuromodulation [22], of eating behaviour [23], suggest that obesity cannot be considered only as a metabolic or behavioural issue, but should be addressed by taking into account brain influences on body weight regulation mechanisms. These findings show the importance of considering the neural basis of nutrition in the treatment of eating disorders, emphasising the need for a multidisciplinary therapeutic approach that integrates neuroscience, psychology and nutrition science to develop more effective and targeted interventions. While NFT has been investigated in populations with binge eating and obesity, demonstrating reductions in pathological eating behaviours [24], the existing evidence is limited to heterogeneous samples that do not include post-bariatric surgery patients. This represents a significant gap, since the clinical profile of post-bariatric patients differs substantially from that of general obese populations. In fact, despite substantial weight loss, many individuals continue to experience maladaptive eating patterns, emotional dysregulation, and vulnerability to relapse, which can undermine long-term surgical outcomes [12]. Moreover, most neurofeedback studies have focused on general measures of food craving or binge eating, without considering the specific psychosocial and neurocognitive challenges that characterise the post-bariatric condition.

Therefore, to our knowledge, no studies have systematically evaluated the use of NFT in this clinical subgroup. Addressing this gap is particularly relevant, as NFT offers the possibility to directly target neural circuits involved in inhibitory control, reward processing, and emotion regulation, which are central to the persistence of emotional eating after surgery.

Based on these premises, the conceptual hypothesis of the present study is that NFT, by enhancing self-regulatory mechanisms through targeted modulation of brain activity, may reduce emotional eating and improve psychological well-being in post-bariatric surgery patients.

## 2. Materials and Methods

### 2.1. Partecipants

A total of 36 patients were recruited at the General Surgery Unit of the University Hospital of Messina. All participants had previously undergone bariatric surgery, specifically sleeve gastrectomy, and provided written informed consent before taking part in the study. The sample was divided into two groups: an experimental group (N = 18), which received neurofeedback treatment, and a control group (N = 18). For each participant, data were collected on biographical information, body mass index (BMI), clinical and psychiatric history, the presence or absence of binge eating episodes, and details related to their bariatric procedure. All questionnaires were administered and completed in paper format.

Inclusion criteria required that participants were eligible for bariatric surgery according to clinical guidelines, had already undergone sleeve gastrectomy, did not present current episodes of binge eating, and reported emotional eating behaviours in the postoperative period. The exclusion criteria included patients age under 18 years, the presence of active binge eating episodes, a body mass index (BMI) below 30, and medical or organic conditions that could interfere with adherence to the neurofeedback protocol.

This study was conducted in accordance with the Decla ration of Helsinki and approved by the Ethics Committee at the University Hospital of Messina (Prot. 68/16 approved 1 August 2016). In addition, this study was conducted according to the diagnostic and therapeutic procedures for the multidisciplinary clinical management of obesity (PDTA No. 2755 approved DG 1261/20 December 2024).

### 2.2. Neurofeedback Protocol Used in the Study

The neurofeedback protocol adopted in the study was based on alpha-theta training. Brain activity was recorded via two electrodes placed in the following leads: left central occipital (C3-O1) and right central occipital (C4-O2). The positioning of the electrodes was chosen to target both the sensorimotor (central) and visual (occipital) cortical regions. The sensorimotor cortex contributes to self-regulatory control and habitual motor patterns, which are potentially relevant in modulating orofacial and eating behaviours, while the occipital areas are involved in the visual processing of signals including food and body image. Neurofeedback targeting alpha-theta activity in these regions can improve emotional regulation and inhibitory control over food-related stimuli, promoting adaptive eating behaviours [25]. The treatment was conducted for a total of 10 sessions, each lasting 30 min, on a weekly basis, for a total duration of two months and two weeks. The sessions took place in a quiet and distraction-free environment, using the Encephalan Main ABP-26 with Rehacor software 221 (Medicom MTM Ltd., Taganrog, Russia). To ensure adequate conductivity, an electrically conductive gel was applied to the participants’ skin. The protocol employed a frequency band between 6 and 9 Hz, with the aim of increasing alpha and theta waves in the open-eye condition. During the training, patients were presented with visual stimuli (geometric shapes, diagrams, graphs, linear distortions, pictures, slides and videos) and auditory stimuli (audio fragments, natural sounds, voice information and sound distortions). In addition, audio and video feedback messages allowed participants to monitor their progress in real time. At the end of each session, the electrodes and cap were removed. During the study, patients were assessed at two points in time: one month after surgery (T0) and after 10 neurofeedback sessions (T1). All 18 participants demonstrated high adherence to the protocol, completing it regularly without any dropouts and attending all scheduled sessions in full.

### 2.3. Measures

#### 2.3.1. Eating Disorder Inventory (EDI)

The Eating Disorder Inventory (EDI) [26] is an instrument used to assess the main psychological characteristics associated with eating disorders. The scale is not intended for the specific diagnosis of an eating disorder, but rather for the evaluation of traits considered significant for understanding such conditions. The instrument consists of 64 items divided into eight subscales: the first three examine attitudes and behaviours related to weight, eating, and body image, while the remaining five assess broader psychological traits that are clinically relevant to eating disorders. Participants respond to each item using a 6-point Likert scale, ranging from always, usually, often, sometimes, rarely, to never. These responses are then recoded into a 4-point scale (0–3), where 0 is assigned to the three least symptomatic responses, and 3 represents the most symptomatic response. The eight subscales of the EDI are: (1) Drive for Thinness, this subscale measures the excessive desire for thinness, which often leads to restrictive and unhealthy weight-loss behaviour. (2) Bulimia, this subscale assesses behaviours related to bulimia nervosa, including episodes of binge eating and subsequent compensatory behaviours such as self-induced vomiting. It measures the frequency and intensity of binge periods and the level of anxiety or shame related to these behaviours. (3) Body Dissatisfaction, this subscale measures the degree of dissatisfaction a person feels about their body. Individuals with high scores in this subscale are more likely to perceive their body as imperfect or ‘wrong’. It focuses on feelings of physical and psychological discomfort with various areas of the body and concerns about fitness. (4) Ineffectiveness¸ this subscale explores the sense of ineffectiveness that often accompanies eating disorders. It is a measure of how the person perceives his or her ability to handle stressful situations and achieve goals. (5) Perfectionism refers to the tendency to set unrealistically high standards for oneself and to feel disappointed or anxious when these standards are not met. (6) Interpersonal Distrust, this subscale measures the level of distrust a person has in others and in their social relationships. (7) Interoceptive Awareness, this subscale explores a person’s ability to perceive and interpret physiological body signals, such as hunger, satiety, tiredness and pain; and (8) Maturity Fears this subscale measures fears related to growth and maturation, including physical and psychological changes associated with adulthood.

#### 2.3.2. Body Uneasiness Test (BUT)

The Body Uneasiness Test (BUT) [27]. It is a self-report questionnaire consisting of 71 items, divided into two sections. The first section, BUT-A, consisting of 34 items, assesses aspects such as Weight phobia, evaluate excessive preoccupation with body weight and fear of gaining weight. Body image concerns, measures the level of dissatisfaction with one’s physical appearance, including distorted perceptions of one’s body, feelings of shame and discomfort about specific body parts. Avoidance assesses the avoidance strategies put in place to avoid confronting one’s body. Compulsive self-monitoring measures the tendency to repetitively and obsessively control one’s physical appearance. Depersonalization assesses the sense of alienation from one’s own body, as if it did not belong to the person or was detached from them. The second part, BUT-B, consisting of 37 items, instead explores specific concerns related to specific body areas or functions. In the present study, only Part A of the Body Uneasiness Test (BUT) was administered.

However, the Binge Eating Scale (BES) was used solely as a screening tool to confirm the exclusion criteria and was therefore not considered in the statistical analyses.

The psychodiagnostic assessment was administered before and after the neurofeedback intervention.

### 2.4. Statistical Analysis

The collected data were analysed using descriptive and inferential statistical techniques. Considering the small sample size and the non-normal distribution of the variables, a non-parametric approach was adopted. Differences between the clinical group and the control group at baseline (T0) and after treatment (T1) were examined using the Mann–Whitney U test for independent samples; the within-group differences between the baseline and final time of the study were assessed by the Wilcoxon rank sum test. In addition, a Cohen’s statistic was applied to measure the magnitude of the treatment effect: the effect size was considered small when it was lower than 0.50, moderate when it ranged from 0.50 to 0.79, and large when it was 0.80 or greater. A *p*-value < 0.05 was considered indicative of statistical significance. Analyses were conducted using Statistical Package for the Social Sciences (SPSS) 25.0 software (SPSS Inc., Chicago, IL, USA).

## 3. Results

The clinical and demographic characteristics of the sample are reported in Table 1. The comparison between the clinical and control groups revealed statistically significant differences. Following neurofeedback treatment, patients in the clinical group showed a significant reduction in eating disorder symptoms. Improvements were evident both in the global questionnaire scores and in specific dimensions assessed by the Eating Disorder Inventory (EDI) and the Body Uneasiness Test (BUT). For the EDI, statistically significant improvements were observed in drive for thinness (*p* = 0.023; d = 0.51), bulimia (*p* = 0.008; d = 0.92), body dissatisfaction (*p* = 0.015; d = 0.52), ineffectiveness (*p* = 0.002; d = 0.89), perfectionism (*p* = 0.006; d = 0.70), interpersonal distrust (*p* = 0.008; d = 0.82), and interoceptive awareness (*p* = 0.001; d = 0.91). No significant changes were found for maturity fear (*p* = 0.191). For the BUT, significant improvements were found in weight phobia (*p* = 0.041; d = 0.84), body image concern (*p* = 0.039; d = 0.90), avoidance (*p* = 0.027; d = 0.83), compulsive self-monitoring (*p* = 0.013; d = 0.83), and depersonalisation (*p* = 0.033; d = 0.85). Within the neurofeedback group, comparing baseline (T0) and post-treatment (T1) scores, significant changes emerged in the global EDI score (*p* = 0.008; d = 0.68) as well as in its subscales: drive for thinness (*p* = 0.008; d = 0.70), bulimia (*p* = 0.008; d = 0.81), body dissatisfaction (*p* = 0.011; d = 0.69), ineffectiveness (*p* = 0.008; d = 0.89), perfectionism (*p* = 0.008; d = 0.72), interpersonal distrust (*p* = 0.008; d = 0.88), and interoceptive awareness (*p* = 0.008; d = 0.88). Significant improvements were also confirmed for the BUT total score and its subscales: weight phobia (*p* = 0.008; d = 0.77), body image concern (*p* = 0.008; d = 0.77), avoidance (*p* = 0.018; d = 0.58) and compulsive self-monitoring (*p* = 0.021; d = 0.63) (Table 2; Figure S1).

## 4. Discussion

This study explores the potential impact of bariatric surgery on eating behaviour and the effectiveness of its combination with neurofeedback. Although the literature suggests that surgery may already induce improvements in eating-related symptoms [28,29,30], the inclusion of a control group was crucial to assess whether the observed improvements in EDI and BUT scores were specifically attributable to the addition of neurofeedback. To date, there are no studies in the literature examining the use of neurofeedback after bariatric surgery to improve emotional eating. However, several studies have separately explored the effects of neurofeedback and bariatric surgery on eating behaviour and psychological symptoms [25,31,32,33,34]. The potential of neurofeedback to improve dysfunctional eating behaviours was explored in an initial literature review [35]. The authors highlighted that EEG-neurofeedback may represent a promising therapeutic option in this area, although the available literature was still limited. A notable contribution to this line of research is a study particularly relevant to the evaluation of neurofeedback in the post-bariatric surgery context [36], which is particularly relevant for the evaluation of the use of neurofeedback in the post-bariatric surgery period. In this study, the effectiveness of neurofeedback was examined in a sample of women with subclinical binge eating disorder. The 75 participants were divided into three groups: one subjected to neurofeedback, one to mental imagery and a control group. The results showed that only the participants treated with neurofeedback reported a significant reduction in binge eating episodes, with effects that persisted even three months after treatment. Further support for these findings is provided by a study involving 38 overweight participants who underwent a single-day training session consisting of six up-regulation trials and passive visualisation [37]. The results showed changes in the brain areas involved, with an impact on eating behaviour. The effects observed with NFT can also be interpreted from a neurobiological point of view: alpha-theta training may modulate the activity of the dorsolateral prefrontal cortex and anterior cingulum, key areas of inhibitory control and food regulation [18,22,37]. Furthermore, the reduction in dysfunction in the mesolimbic dopaminergic circuit, implicated in emotional eating behaviours, could contribute to better control of impulses and food-related stimuli [38]. Similarly, a study involving six participants who underwent ten neurofeedback sessions using a near-infrared haemencephalogram (NIRHEG) reported improvements in eating behaviour and appetite control, as well as significant weight loss [21]. These results suggest that a cycle of ten sessions can have lasting effects of up to six months, which could be further extended with maintenance sessions. Regarding changes in emotional eating profiles following bariatric surgery, the literature suggests positive results. A recent study [39] compared three different bariatric surgical procedures: laparoscopic adjustable gastric banding (LAGB), Roux-en-Y gastric bypass (RYGB) and biliopancreatic diversion (BPD). The overall results indicated that all three surgical procedures lead to a reduction in emotional eating. Furthermore, other results [40] indicate that the severity of problematic eating behaviours decreased significantly after bariatric surgery and remained below preoperative levels during the follow-up period. However, other neuromodulatory techniques have been explored to address maladaptive eating behaviours. Transcranial magnetic stimulation (TMS), particularly when applied to the dorsolateral prefrontal cortex (DLPFC), has been shown to modulate food cravings and consumption. For example, high-frequency rTMS targeting the left DLPFC reduced cue-induced food cravings and binge episodes in individuals with bulimic-type disorders [41]. More broadly, meta-analytic evidence indicates that non-invasive brain stimulation over the DLPFC produces small-to-moderate reductions in both food craving and consumption [42]. Although the mechanisms differ—TMS delivers external stimulation to cortical networks, whereas neurofeedback promotes self-regulation through operant conditioning—both approaches target neural circuits implicated in inhibitory control and emotion regulation. Future studies could investigate whether combining or sequencing these techniques might maximise behavioural improvements in post-bariatric surgery patients.

Beyond neuromodulatory interventions, psychotherapeutic approaches remain essential for optimising long-term outcomes in post-bariatric surgery patients. Beaulac et al. [43], highlighted that postoperative psychosocial support, particularly CBT, is associated with better adherence to dietary recommendations, greater weight loss maintenance, and improved psychological well-being in bariatric populations. Integrating neurofeedback with psychotherapy could therefore provide synergistic benefits, with neuromodulation enhancing neurophysiological self-regulation and psychotherapy consolidating these gains through cognitive restructuring, coping skills training, and behaviour change strategies. In addition to efficacy considerations, it is necessary to consider the clinical feasibility of integrating neurofeedback therapy (NFT) into post-bariatric follow-up programmes. Treatment can be delivered in outpatient settings, with cycles of 8–12 sessions spread over 2–3 months, in parallel with the periodic check-ups already scheduled. This approach would facilitate its inclusion without significantly increasing the care burden for patients. Furthermore, its non-invasive and generally well-tolerated nature makes NFT compatible with other forms of psychological and nutritional support. Some reviews have reported good acceptability and low drop-out rates from treatment in various clinical settings [44,45]. Looking ahead, the development of portable systems and simplified protocols could facilitate its application in community settings or via telerehabilitation, increasing accessibility. However, significant challenges remain: the need for trained personnel, the cost of equipment, and the lack of standardised protocols. For effective implementation, it will therefore be essential to define selection criteria, assess economic sustainability, and conduct randomised confirmatory studies.

## 5. Conclusions

The results of this study support the initial hypothesis that NFT, applied as a complementary intervention in patients undergoing bariatric surgery, was associated with a significant reduction in various aspects of eating symptoms and an improvement in some indicators of body image. These findings, although limited by the small sample size, suggest that NFT may represent a non-invasive and potentially effective option for addressing residual emotional eating behaviour and related psychological problems in the post-operative period. From a clinical perspective, the plausibility of the results is supported by their consistency with preliminary literature on the use of NFT in eating disorders, which highlights how neurophysiological self-regulation can positively influence dysfunctional eating behaviours and body perception. This suggests that NFT may represent a non-invasive and potentially effective option for integration into post-operative programmes, in synergy with psychotherapeutic interventions and behavioural strategies, in order to maximise the maintenance of results and promote long-term psychological well-being. From an operational point of view, healthcare professionals involved in the multidisciplinary management of bariatric patients are advised to consider integrating neurofeedback into follow-up programmes, particularly in individuals who, despite the initial success of surgery, experience difficulties with emotional regulation or body dissatisfaction. The application should be carried out in synergy with psychotherapeutic interventions and behavioural strategies in order to maximise the maintenance of results and promote lasting psychological well-being. Randomised studies with larger samples and long-term follow-ups are needed to confirm the effectiveness and define standardised protocols for the use of NFT in this context

## 6. Limitations

This study has several limitations that should be considered when interpreting the findings. First, the small sample size reduces both statistical power and generalisability; as the study is still ongoing, the collection of additional data will allow for more robust analyses. Second, the lack of randomisation—since allocation to the neurofeedback group was based on participants’ consent—may have introduced a selection bias, and results should therefore be interpreted with caution.

Moreover, the absence of an active placebo group and the failure to control for concurrent interventions (e.g., psychological support) limit the possibility of drawing firm conclusions about the specific effects of neurofeedback. Another limitation concerns the magnitude of reductions observed in some dimensions, such as the bulimia subscale of the EDI. Although these changes may reflect protocol efficacy and participants’ adherence, the influence of non-specific factors (e.g., placebo effect, motivational impact of study participation) cannot be excluded.

Finally: the study did not translate the reductions observed in EDI and BUT subscales into concrete clinical indicators (e.g., nutritional adherence, relapse reduction, BMI), which restricts the practical applicability of the results. Future studies should address these issues by adopting randomised controlled designs, including active control groups, systematically monitoring concomitant interventions, and linking psychometric changes to clinical outcomes.

## Figures and Tables

**Table 1:** The clinical and demographic characteristics of the sample.

	Neurofeedback Group (N = 18)	Control Group (N = 18)
Gender (M; F)	10; 8	9; 9
Age (mean ± SD)	45.22 ± 11.2	42.22 ± 11.2
Schooling (n, %)	Primary school: 2 (11.1%) Middle school: 12 (66.7%) High school: 4 (22.2%)	Primary school: 3 (16.7%) Middle school: 9 (50%) High school: 6 (33.3%)
Psychiatric history-Psychopharmacological treatment (n, %)	4 (22.2%)	2 (11.1%)

**Table 2:** Descriptive statistics and differences in the Wilcoxon test between time T0 and time T1 of the NFT and Mann–Whitney U test between NFT and Control group.

	Neurofeedback Group (N = 18)						Control Group (N = 18)				NFT vs. Control Group		
	T0		T1		T0 vs. T1		T0		T1		T0	T1	
	Mean (S.D)	IC 95%	Mean (S.D)	IC 95%	*p*	Cohen d	Mean (S.D)	IC 95%	Mean (S.D)	IC 95%	*p*	*p*	Cohen d
EDI													
Total score	44.11 (15)	[32.89–55.33]	22.9 (6.5)	[17.90–27.88]	0.008 *	0.68	41.12 (12)	[41.55–62.90]	39.2 (9.3)	[25.33–49.75]	0.235	0.012 *	0.71
Drive for thinness	55 (21.4)	[38.25–71.23]	17.86 (15.2)	[6.18–29.55]	0.008 *	0.70	51 (21.4)	[44.81–75.80]	38.23 (18.2)	[16.97–48.56]	0.371	0.023 *	0.51
Bulimia	35.03 (15.1)	[23.37–46.70]	3.16 (4.7)	[−0.485–6.82]	0.008 *	0.81	36.03 (11.2)	[28.77–46.68]	34.13 (7.3)	[6.90–22.42]	0.435	0.008 *	0.92
Body dissatisfaction	53.74 (20.5)	[37.98–69.51]	18.92 (15.1)	[7.31–30.54]	0.011 *	0.69	55.72 (25.1)	[47.67–76.72]	35.22 (11.2)	[22.19–61.55]	0.289	0.015 *	0.52
Ineffectiveness	50.26 (16.6)	[37.49–63.04]	1.85 (3.8)	[−1.045–4.76]	0.008 *	0.89	56.12 (15.6)	[43.43–70.70]	53.99 (17.9)	[11.86–31.45]	0.126	0.002 *	0.89
Perfectionism	51.77 (9.6)	[44.34–59.19]	19.02 (19.9)	[3.71–34.33]	0.008 *	0.72	53.88 (11.2)	[47.11–60.94]	52.09 (12.1)	[14.34–35.67]	0.366	0.006 *	0.70
Interpersonal distrust	50.06 (11.6)	[41.01–59.01]	11.64 (8.3)	[5.28–18.00]	0.008 *	0.88	51.06 (15.6)	[43.75–52.32]	49.46 (16.3)	[19.49–30.72]	0.568	0.008 *	0.82
Interoceptive awareness	38.11 (14.3)	[27.11–49.11]	0.73 (1.4)	[−0.385–1.85]	0.008 *	0.88	44.12 (17.3)	[30.27–51.28]	44.01 (13.5)	[7.11–18.59]	0.098	0.001 *	0.91
Maturity fear	34.33 (20.6)	[18.45–50.21]	23.61 (11.6)	[14.70–32.52]	0.109	0.30	35.11 (18.6)	[24.41–47.00]	29.61 (16.6)	[17.99–41.55]	0.479	0.191	0.20
BUT													
Total Score	66.11 (38.1)	[36.85–95.37]	7.66 (6.0)	[3.07–12.26]	0.008 *	0.73	64.15 (39.1)	[54.35–98.76]	62.66 (33.9)	[18.95–49.03]	0.315	0.006 *	0.74
Weight Phobia	2.54 (1.2)	[1.60–3.48]	0.37 (0.3)	[0.13–0.615]	0.008 *	0.77	4.541 (1.0)	[1.57–3.15]	3.9 (1.5)	[0.64–1.84]	0.198	0.041 *	0.84
Body image concern	2.74 (1.4)	[1.66–3.82]	0.271 (0.2)	[0.085–0.46]	0.008 *	0.77	3.74 (1.5)	[2.51–4.27]	4.01 (1.2)	[0.57–1.55]	0.297	0.039 *	0.90
Avoidance	1.31 (1.2)	[0.37–2.25]	0.055 (0.2)	[−0.073–0.184]	0.018 *	0.58	1.1 (1.7)	[0.98–2.56]	1.01 (1.2)	[0.13–1.66]	0.789	0.027 *	0.48
Compulsive self-monitoring	1.09 (0.8)	[0.49–1.68]	0.17 (0.2)	[0.02–0.30]	0.021 *	0.63	1.55 (0.8)	[0.60–1.58]	1.87 (0.8)	[0.06–1.13]	0.685	0.013 *	0.83
Depersonalization	1.33 (1.4)	[0.28–2.39]	0.177 (0.2)	[0.016–0.34]	0.058	0.51	1.99 (0.9)	[1.54–2.71]	1.87 (0.7)	[0.29–1.80]	0.590	0.033 *	0.85

Abbreviations: EDI = Eating Disorder Inventory; BUT = Body Uneasiness Test; * *p* < 0.05.

## Data Availability

The data presented in this study are available on request from the authors who reserve the right to make them available.

## Supplementary Materials

The following supporting information can be downloaded at: https://www.mdpi.com/article/10.3390/jpm15100454/s1, Figure S1: Boxplot Neurofeedback Group (T0 vs. T1)—BUT and EDI subscale.

## Abbreviations

The following abbreviations are used in this manuscript:

NFT	Neurofeedback Training
EDI	Eating Disorder Inventory
BUT	Body Uneasiness Test
EE	Emotional Eating
BMI	Body Mass Index
