# Supplementary material for: Evaluating the Efficacy of Neurofeedback in Post-Bariatric Surgery Patients: A Pilot Study
Source: J Pers Med. 2025 Sep 29;15(10):454. doi: 10.3390/jpm15100454 (PMC12565648; doi:10.3390/jpm15100454)
Supplement: Supplementary file 1 [file jpm-15-00454-s001.zip › jpm-3775805-supplementary.pdf]

Supplementary Material

Supplementary Figure S1: Boxplot Neurofeedback Group (T0 vs T1) – BUT and EDI subscales

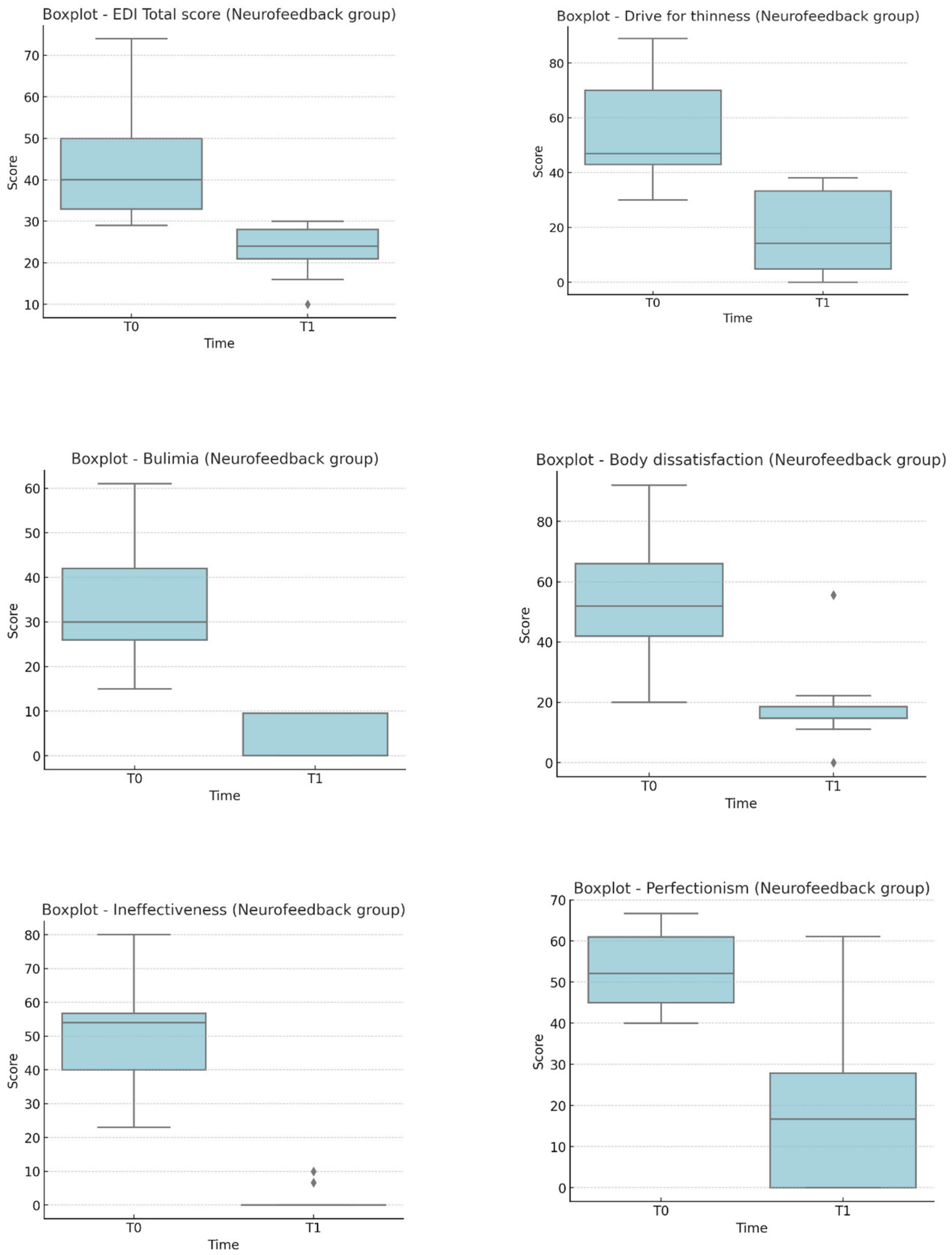

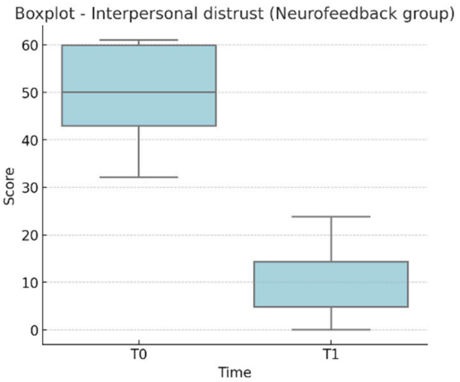
Boxplot - Interpersonal distrust (Neurofeedback group)
Score
Time
T0
T1

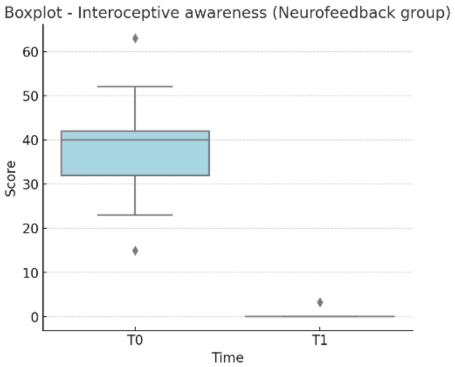
Boxplot - Interoceptive awareness (Neurofeedback group)
Score
Time
T0
T1

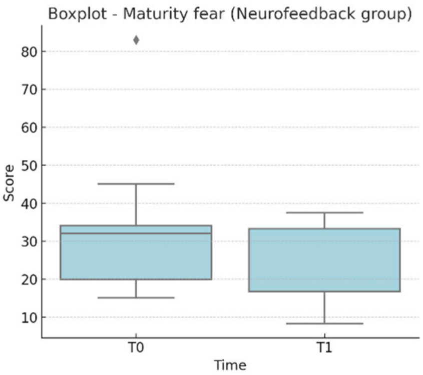
Boxplot - Maturity fear (Neurofeedback group)
Score
Time
T0
T1

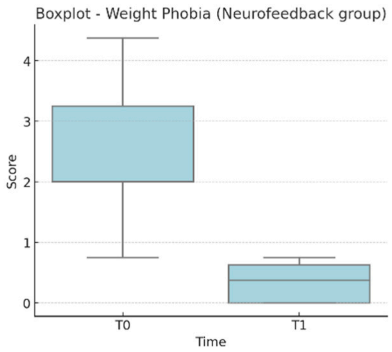
Boxplot - Weight Phobia (Neurofeedback group)
Score
Time
T0
T1

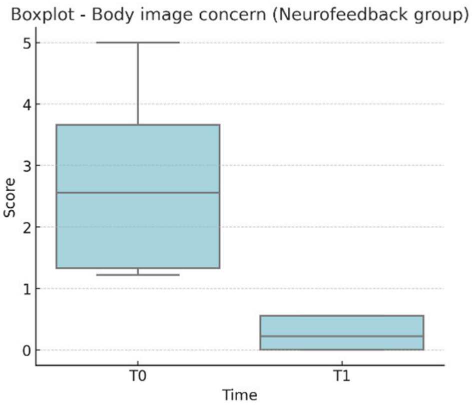
Boxplot - Body image concern (Neurofeedback group)
Score
Time
T0
T1

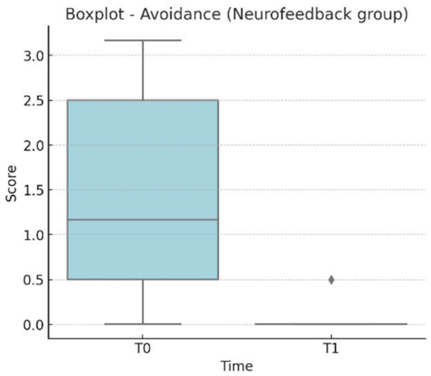
Boxplot - Avoidance (Neurofeedback group)
Score
Time
T0
T1

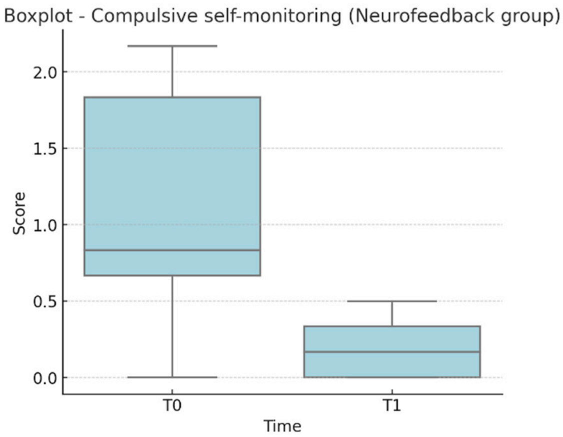
Boxplot - Compulsive self-monitoring (Neurofeedback group)
Score
Time
T0
T1

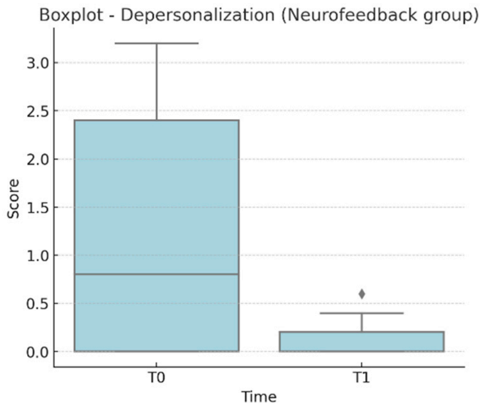
Boxplot - Depersonalization (Neurofeedback group)
Score
Time
T0
T1

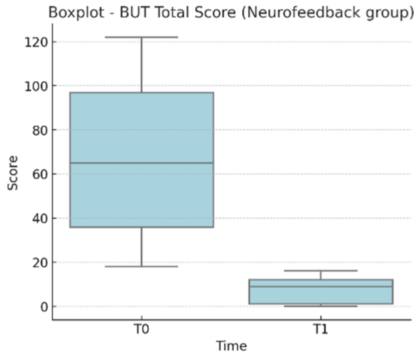

Scatter plot Neurofeedback Group (T0 vs T1) – BUT and EDI subscale

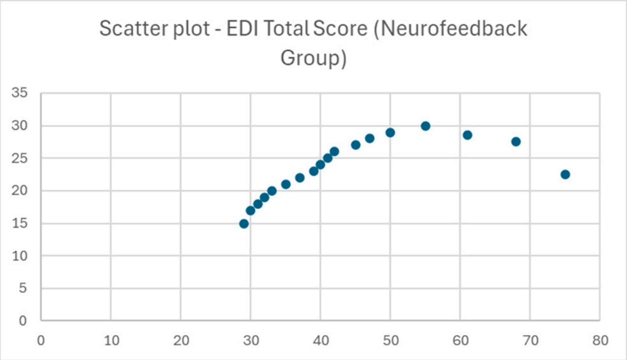

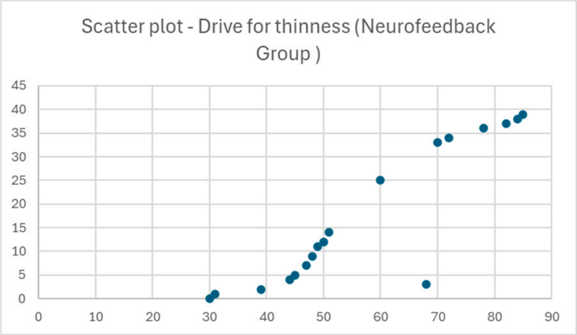

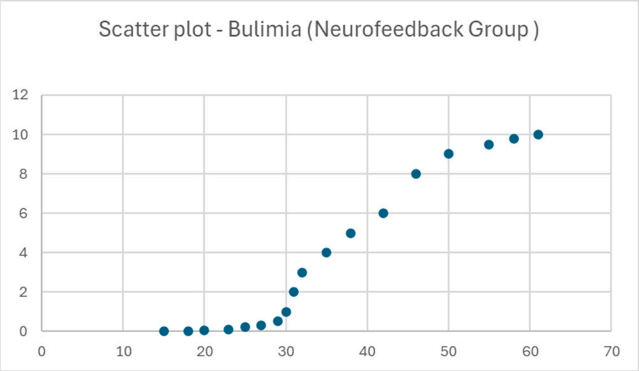

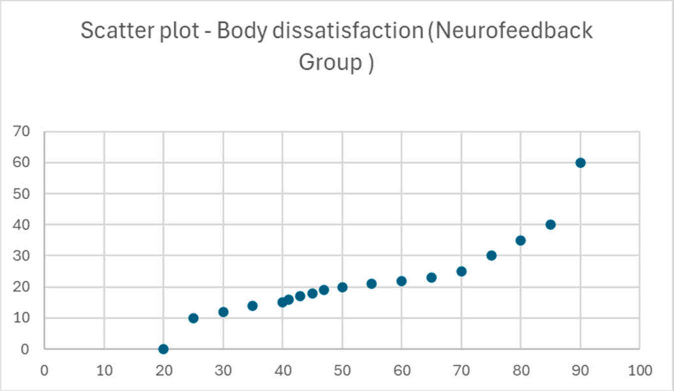

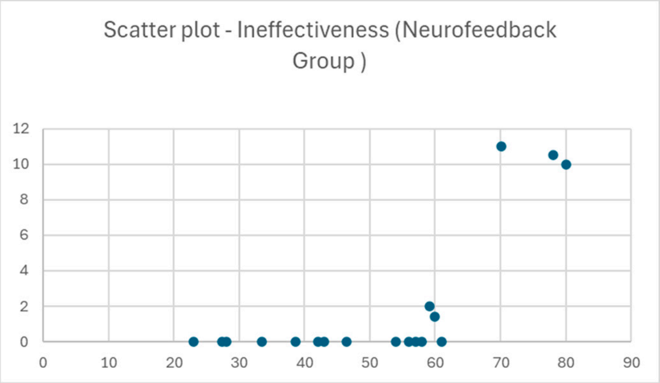

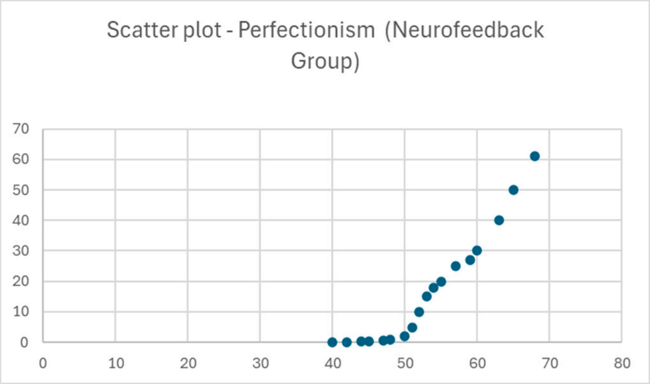

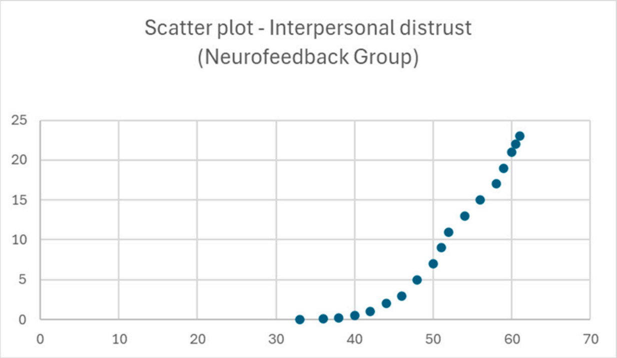
Scatter plot - Interpersonal distrust
(Neurofeedback Group)
25
20
15
10
5
0
0
10
20
30
40
50
60
70

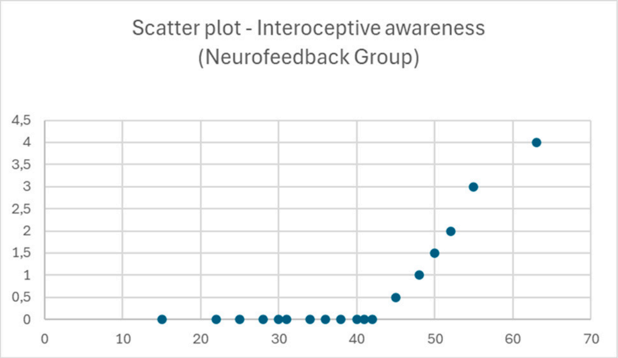
Scatter plot - Interoceptive awareness
(Neurofeedback Group)
4,5
4
3,5
3
2,5
2
1,5
1
0,5
0
0
10
20
30
40
50
60
70

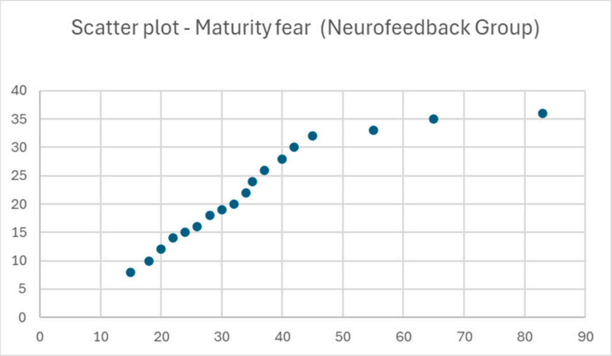
Scatter plot - Maturity fear (Neurofeedback Group)
40
35
30
25
20
15
10
5
0
0
10
20
30
40
50
60
70
80
90

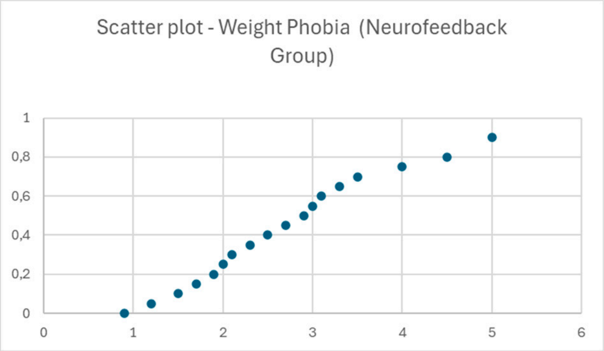
Scatter plot - Weight Phobia (Neurofeedback
Group)
1
0,8
0,6
0,4
0,2
0
0
1
2
3
4
5
6

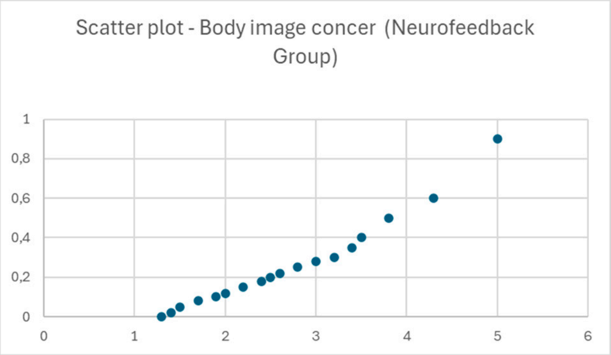
Scatter plot - Body image concer (Neurofeedback
Group)
1
0,8
0,6
0,4
0,2
0
0
1
2
3
4
5
6

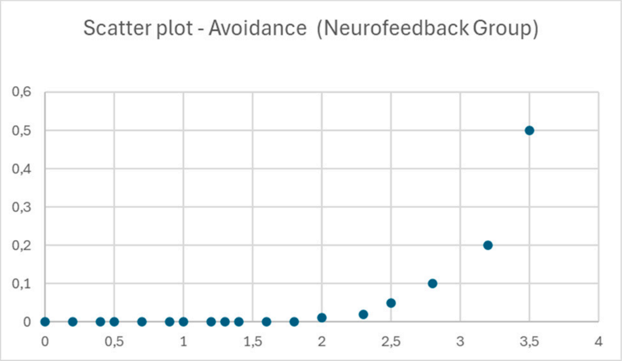
Scatter plot - Avoidance (Neurofeedback Group)
0,6
0,5
0,4
0,3
0,2
0,1
0
0
0,5
1
1,5
2
2,5
3
3,5
4

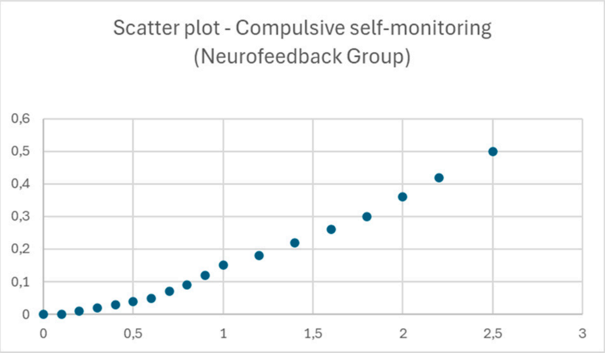
Scatter plot - Compulsive self-monitoring
(Neurofeedback Group)
0,6
0,5
0,4
0,3
0,2
0,1
0
0
0,5
1
1,5
2
2,5
3

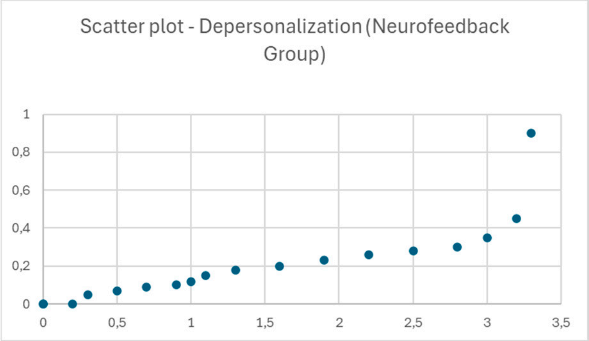
Scatter plot - Depersonalization (Neurofeedback
Group)
1
0,8
0,6
0,4
0,2
0
0
0,5
1
1,5
2
2,5
3
3,5

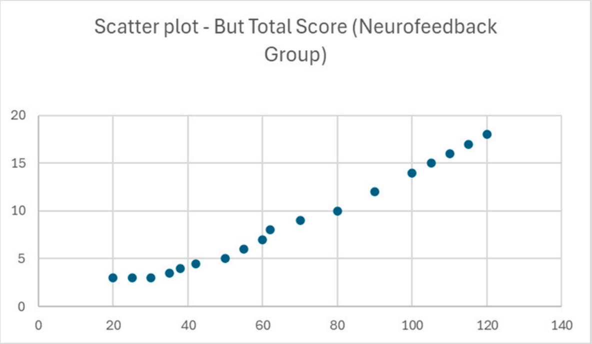

Skewness and Kurtosis of Neurofeedback Group (T0 vs T1) – BUT and EDI subscales

Neurofeedback Group (n.18)				
	T0		T1	
	Skewness	Kurtosis	Skewness	Kurtosis
**EDI**				
**Total score**	1,020	,944	-1,025	,642
**Drive for thinness**	,449	-1,177	,239	-1,607
**Bulimia**	,562	-,662	,857	-1,714
**Body dissatisfaction**	,315	,803	1,952	5,410
**Ineffectiveness**	-,012	,558	1,822	2,080
**Perfectionism**	,323	-1,380	1,121	1,473
**Interpersonal distrust**	-,625	-1,215	-,343	-,924
**Interoceptive awareness**	,117	,279	1,620	,735
**Maturity fear**	1,825	4,132	,127	-2,062
**BUT**				
**Total Score**	,363	-1,467	-,279	-1,448
**Weight Phobia**	,363	-,785	-,185	-1,678
**Body image concern**	,524	-1,090	,096	-1,811
**Avoidance**	,517	-1,471	3,00	9,00
**Compulsive self-monitoring**	-,032	-1,453	,690	-,800
**Depersonalization**	,402	-1,836	1,094	,611
